# Rabson-Mendenhall syndrome presented as severe acanthosis nigricans in an infant harboring novel mutations in the INSR gene: a case report

**DOI:** 10.3389/fped.2025.1511429

**Published:** 2025-04-16

**Authors:** Shi Yan, Yu Sheng, Hai-zhen Hui, Chun-yan Zhou, De-ming Liu

**Affiliations:** ^1^ Chongqing Clinical Research Center for Dermatology, Chongqing Key Laboratory of Integrative Dermatology Research, Key Laboratory of External Therapies of Traditional Chinese Medicine in Eczema, Department of Dermatology, Chongqing Traditional Chinese Medicine Hospital/The First Affiliated Hospital of Chongqing College of Traditional Chinese Medicine, Chongqing, China; ^2^ General Surgery, Chongqing Traditional Chinese Medicine Hospital/The First Affiliated Hospital of Chongqing College of Traditional Chinese Medicine, Chongqing, China

**Keywords:** INSR gene, Rabson-Mendenhall syndrome, acanthosis nigricans, case report, acanthosis nigricans (AN)

## Abstract

**Background:**

Rabson-Mendenhall Syndrome (RMS), a rare hereditary form of insulin resistance, is marked by severe hyperinsulinemia and early-onset acanthosis nigricans (AN) during childhood.

**Case presentation:**

A case of a 15-month-old girl was reported, presenting with widespread acanthosis nigricans, growth retardation, dysmorphic facial features, and hypertrichosis. Laboratory results indicated fasting hypoglycemia and hyperinsulinemia, while her oral glucose tolerance test (OGTT) remained normal. Whole-exome sequencing revealed two novel mutations in the insulin receptor gene (INSR): a c.3392 C > G missense/frameshift mutation in exon 19 and a c.4007_4010delAGAG deletion in exon 22.

**Conclusion:**

Acanthosis nigricans (AN) can serve as a clinical marker that strongly suggests underlying metabolic syndromes, making genetic analysis essential for confirming the diagnosis.

## Background

Acanthosis nigricans (AN) is a reactive skin condition that is strongly associated with insulin resistance and is recognized as a morphological indicator of metabolic syndrome (1). The phenotypic severity of insulin resistance syndromes resulting from mutations in the INSR gene is determined by the extent of INSR dysfunction (2). Donohue syndrome (DS), also known as leprechaunism, represents the most severe form, while type A insulin resistance syndrome is characterized by milder clinical manifestations. Rabson-Mendenhall syndrome (RMS) is an intermediate form between these conditions (2). In this report, we describe a Chinese female patient with RMS who carries two novel mutations in the INSR gene and presents with generalized acanthosis nigricans and hyperinsulinism.

## Case presentation

**Figure 1:**
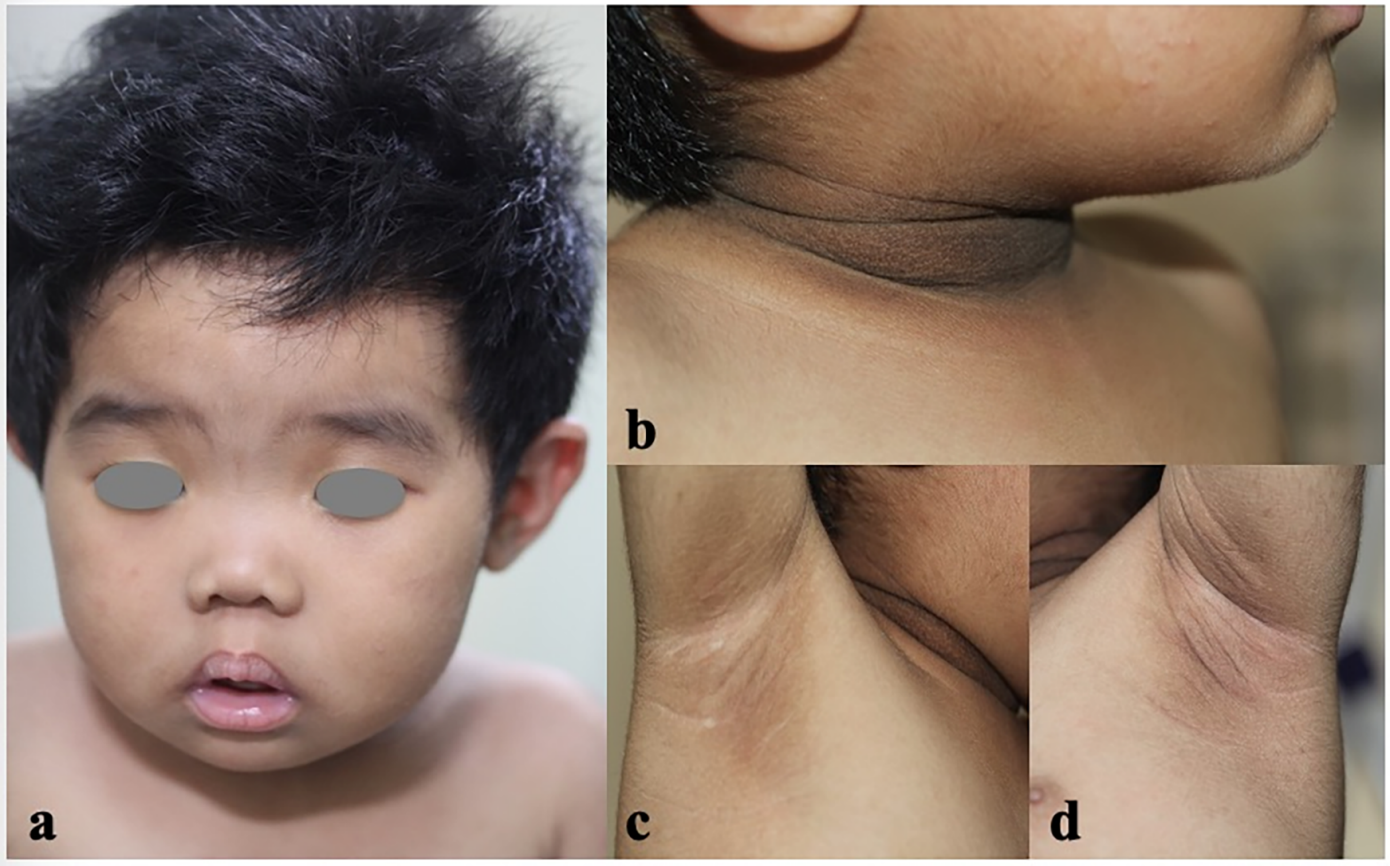
The clinical features of the patient. Coarse face with large ears, saddle nose, thick lips, hypertrichosis **(a)**, and severe acanthosis nigricans of the neck and axillae **(b–d)** were noted.

**Figure 2:**
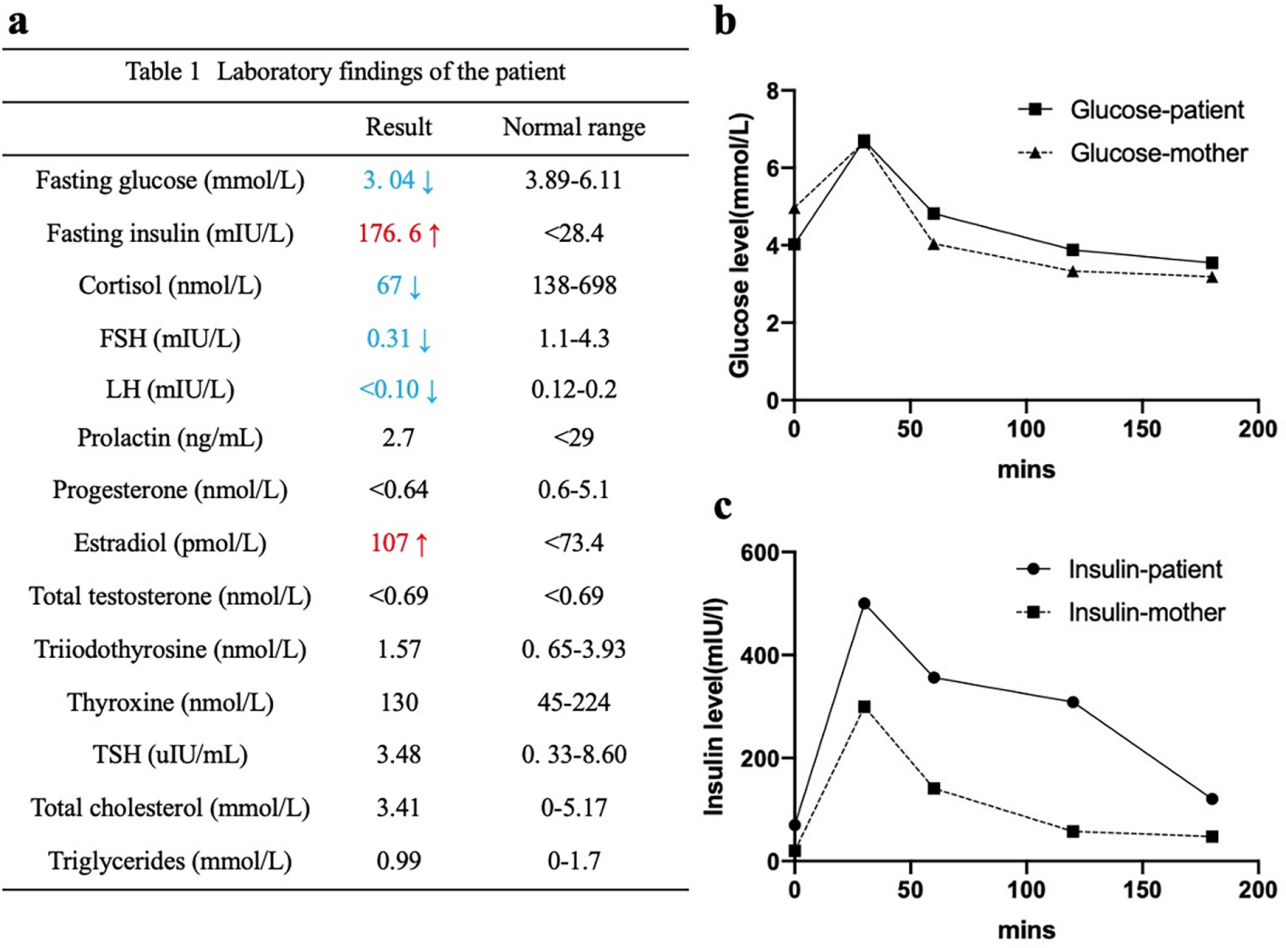
Laboratory findings of the patient. Hyperinsulinemia **(a,c)**, fasting hypoglycemia **(a)**, impaired glucose tolerance **(b,c)** were detected.

A 15-month-old female was referred to our dermatology outpatient clinic due to severe acanthosis nigricans, which had developed over the past nine months. She was the first and only child of a non-consanguineous family and had no family history of related conditions. The patient was delivered by cesarean section at 41 weeks of gestation, with a low birth weight of 2000 grams (<3rd percentile) and a length of 48 cm (<25th percentile). Growth retardation was noted, as she consistently demonstrated low weight and length (<3rd percentile) at 3 months (4.9 kg, 57 cm), 6 months (6.3 kg, 63.5 cm), and 12 months (8 kg, 71 cm). Her developmental milestones were otherwise normal. On examination, she presented with severe acanthosis nigricans on the neck, axillae, and external genitalia, along with hypertrichosis, coarse facial features, large ears, a saddle nose, and thick lips (Figure 1). Laboratory tests revealed normal liver and renal function, lipid profile, thyroid hormone levels, and electrolytes. However, her fasting glucose was low (3.04 mmol/L), while her fasting insulin was markedly elevated (176.6 mIU/L) (Figure 2a). An oral glucose tolerance test (OGTT) (1.75 g/kg) and glycated hemoglobin (GHb) test were performed, both of which returned normal results (Figures 2a–c) (3). Additionally, abdominal and pelvic ultrasound, as well as brain and pituitary MRI, revealed no abnormalities.

**Figure 3:**
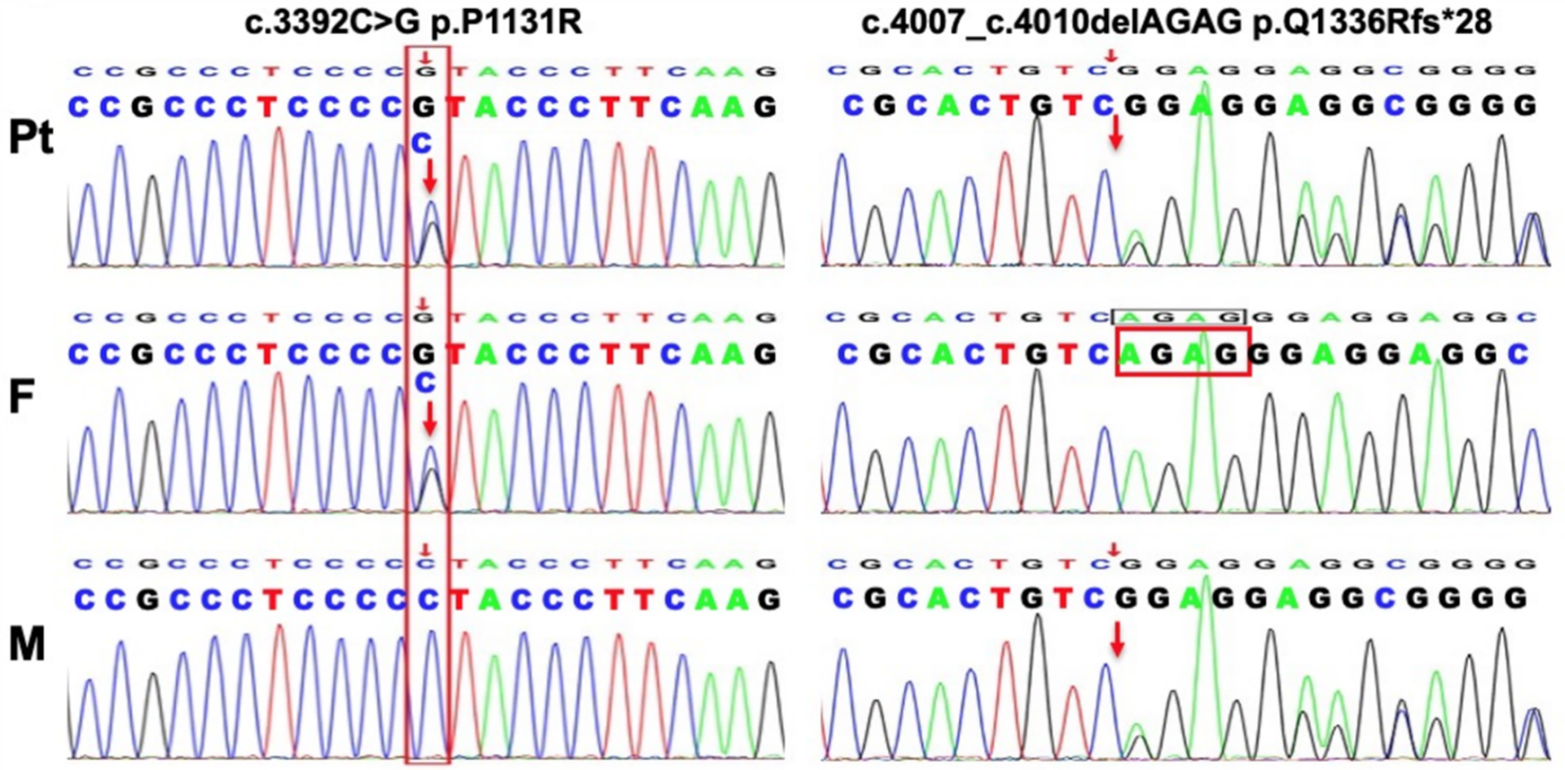
Genetic analysis of this patient. Tow novel mutations in the INSR gene identified in the family members.

Given the clinical and laboratory findings, an inherited insulin resistance syndrome was suspected. After obtaining informed consent from the parents, whole-exome sequencing identified a novel compound heterozygous mutation in the INSR gene: p.P113R (c.3392C > G) inherited from the father and p.Q1336Rfs*28 (c.4007_4010delAGAG) from the mother (Figure 3). Following the diagnosis of Rabson-Mendenhall syndrome (RMS), lifestyle modifications and healthy eating habits were recommended. At the 3-year follow-up, the patient maintained a stable metabolic status, although her clinical features remained unchanged.

## Discussion

Acanthosis nigricans (AN) commonly presents as dark, velvety hyperpigmentation, typically involving the neck, flexural regions, and intertriginous areas. This reactive cutaneous change is closely associated with hyperinsulinemia, insulin resistance, obesity, endocrinopathies, or malignancies, particularly gastrointestinal adenocarcinoma (1). AN associated with impaired glucose tolerance or hyperinsulinemia is commonly observed in patients with insulin resistance syndromes (4). Rabson-Mendenhall syndrome (RMS), one of the insulin resistance syndromes, typically presents with extensive acanthosis nigricans (AN) that has an early onset in childhood, along with hypertrichosis, dysmorphic facial features, growth retardation, hyperinsulinemia, and preprandial hypoglycemia. All the clinical manifestations and laboratory findings of this patient are consistent with RMS. Based on the evidence, AN is indeed an effective clinical marker for identifying patients susceptible to insulin resistance, especially when accompanied by metabolic dysfunction (1); However, the correlation between acanthosis nigricans (AN) and the degree of insulin resistance remains to be confirmed. Severe insulin resistance is typically caused by mutations in the INSR gene, located on chromosome 19p13.2, which consists of 22 exons and 21 introns (5). The INSR gene encodes the insulin receptor, which consists of two extracellular *α*-subunits and two transmembrane *β*-subunits. The *α*-subunits, encoded by exons 1–11, are responsible for ligand binding, whereas the *β*-subunits, encoded by exons 12–22, regulate tyrosine kinase activity, which is crucial for signal transduction (6). Previous studies (2, 7) have demonstrated that mutations in the FnIII domains of the *α*-subunits can severely impair insulin receptor binding, leading to more severe insulin resistance syndromes. Conversely, mutations in the *β*-subunits typically affect tyrosine kinase activity, hindering insulin-mediated glucose transport, and result in milder clinical presentations. To date, only a few mutations in the INSR gene have been associated with RMS, with compound heterozygous mutations being more prevalent. In this case, sequence analysis of the INSR gene revealed two previously unreported novel mutations in the *β*-subunits, located in exons 19 and 22. The patient's relatively mild phenotype aligns with findings from prior research, supporting the hypothesis that mutations in the *β*-subunits lead to less severe clinical manifestations. Studies (8, 9) suggest that there are no statistically significant differences observed in the clinical characteristics or laboratory findings among patients with varying mutations.

The management of Rabson-Mendenhall syndrome (RMS) focuses on mitigating severe insulin resistance and preventing associated complications. Initial treatment typically involves the use of insulin sensitizers to lower blood glucose and HbA1c levels, but their efficacy often diminishes over time, necessitating dose adjustments and combination therapy. High-dose insulin is commonly required, particularly during episodes of diabetic ketoacidosis (10). To address the common endocrine and metabolic issues in RMS, standard treatments for hypothyroidism, anti-androgen therapies, gonadotropin-releasing hormone (GnRH) agonists, and in some cases, oophorectomy are employed. Additionally, long-standing hyperglycemia can lead to microvascular complications such as retinopathy and nephropathy, which are the primary causes of mortality in RMS patients. Therefore, early monitoring and intervention for these complications are crucial (11). For this patient, lifestyle modifications and healthy eating habits were recommended. At the 3-year follow-up, although her metabolic status remained stable, her clinical features showed no significant improvement. This highlights the challenge of managing Rabson-Mendenhall syndrome, where maintaining metabolic control can be achieved with proper interventions, but the progression of physical manifestations often remains unaffected. Continued close monitoring and adaptive treatment strategies are essential to prevent potential complications and ensure long-term stability.

In conclusion, we report a case of a patient with RMS presenting with extensive acanthosis nigricans (AN) and carrying novel mutations in the INSR gene. As dermatologists, it is crucial to consider inherited metabolic syndromes in patients with extensive AN and metabolic dysfunctions. Early screening for INSR gene mutations can aid in timely and accurate diagnosis.

## Data Availability

The raw data supporting the conclusions of this article will be made available by the authors, without undue reservation.
